# Age-Dependent Risks of Incidence and Mortality of COVID-19 in Hubei Province and Other Parts of China

**DOI:** 10.3389/fmed.2020.00190

**Published:** 2020-04-30

**Authors:** Hongdou Li, Shuang Wang, Fan Zhong, Wuyin Bao, Yipeng Li, Lei Liu, Hongyan Wang, Yungang He

**Affiliations:** ^1^Shanghai Key Laboratory of Medical Epigenetics, Institutes of Biomedical Sciences, Fudan University, Shanghai, China; ^2^International Co-laboratory of Medical Epigenetics and Metabolism, Ministry of Science and Technology, Institutes of Biomedical Sciences, Fudan University, Shanghai, China; ^3^The Institute of Reproduction and Developmental Biology, Obstetrics Gynecology Hospital, Fudan University, Shanghai, China; ^4^Guanghan Research Center of Personalized Healthcare, Shanghai, China

**Keywords:** coronavirus, SARS-CoV-2, COVID-19, incidence risk, mortality risk

## Abstract

The new coronavirus SARS-CoV-2 pandemic of early 2020 poses an enormous challenge to global public health. Coronavirus Disease 2019 (COVID-19) caused by the virus has spread rapidly throughout the world, taking thousands of lives in just over 2 months. It is critical to refine the incidence and mortality risks of COVID-19 for the effective management of the general public and patients during the outbreak. In this report, we investigate the incidence and mortality risks of the infection by analyzing the age composition of 5,319 infected patients, 76 fatal cases, and 1,144,648 individuals of the general public in China. Our results show a relatively low incidence risk for young people but a very high mortality risk for seniors. Notably, mortality risk could be as high as 0.48 for people older than 80 years. Furthermore, our study suggests that a good medical service can effectively reduce the mortality rate of the viral infection to 1% or less.

## Introduction

On January 7, 2020, a new pathogenic virus causing pneumonia was identified in a sample of bronchoalveolar lavage fluid from a patient in Wuhan, Hubei province, China. The pathogen had typical features of the coronavirus family and therefore was classified in the subgenus *Sarbecovirus, Orthocoronavirinae* subfamily (1, 2). This virus has been named “severe acute respiratory syndrome coronavirus 2” (SARS-CoV-2), and the disease it causes has been named “coronavirus disease 2019” (COVID-19). It is the third epidemic coronavirus that has emerged in the human population in the Twenty-first century, following the severe acute respiratory syndrome coronavirus (SARS) outbreak in 2002, and the Middle East respiratory syndrome coronavirus (MERS) outbreak in 2012 (3, 4).

Coronavirus is one of the main causes of human respiratory disease owing to frequent cross-species infections. The emerging virus rapidly became a challenge for global public health due to it spreading through human-to-human transmission. The majority of the earliest COVID-19 patients were linked to the Huanan Seafood Wholesale Market. However, human-to-human transmission has frequently occurred, and the epidemic has been gradually growing (5). As of March 4, 2020, 80,566 laboratory-confirmed cases had been reported in China. Internationally, more than 14,396 cases had been reported in 77 countries (6, 7). The number of infected individuals is far surpassing that of SARS and MERS. SARS-CoV-2 can cause severe and even fatal respiratory diseases, such as acute respiratory distress syndrome. It has been reported that SARS-CoV-2 is more likely to affect older males with comorbidities, suggesting that age, and comorbidity may be risk factors for poor outcomes (8, 9). In China, the reported death rate approached 3% of the total number of COVID-19 patients during February 2020.

At present, information regarding the prevalence and case-fatality for the clinical features and epidemiology of COVID-19 remains scarce. However, a relatively accurate evaluation of incidence and mortality is required to help refine the risk assessment and to ensure that the public and patients are managed in an effective way. Therefore, it is necessary to quantitatively evaluate the risks for individual groups of different ages and genders. In this paper, we report our initial analysis of the public data from local authorities. Our study shows that the incidence risk of COVID-19 might be as low as 0.1 for children, while it could be over 0.9 for 40-year-old adults. Our results also suggest that the mortality risk might be above 0.2 for patients older than 80 years. Notably, the mortality risk was significantly different between patients of Hubei province and those from other parts of China.

## Methods

### Data Preparation

Basic information on COVID-19 cases was released on official websites by the National Health Commission of China and its local branches. We collected data from a total of 6,673 identified cases published before February 22. Based on the completeness of the data, we involved 5,319 cases in our study. All the 5,319 COVID-19 cases were residents outside Hubei Province. A total of 76 fatal cases were included in our analysis. Among the 76 fatal cases, 45 cases were reported as residents of Hubei province, and 31 cases were reported as residents of other parts of China. Epidemiological characteristics such as age, gender, and location were carefully checked to remove missing values or duplicated records. The composition of the age of the general public was obtained from data of 1,144,648 individuals from the General Census of China (2018) (10). The census data were collected from the whole country, including 31 provinces, autonomous regions, and municipalities directly under the Central Government. Ethical approval for this study and written informed consent from the participants of the study were not required in accordance with local legislation and national guidelines.

### Estimating Incidence Risk of COVID-19

We estimated the incidence risk of COVID-19 for different age groups in the general public by a maximum likelihood approach. In this approach, given the age composition of the general public and the incidence risk of different age groups, age composition of COVID-19 cases can be obtained as

$$\begin{array}{l} \text{P}\left(Age_{i}|incidence\right)=\frac{\text{P}\left(incidece|Age_{i}\right)P\left(Age_{i}\right)}{\underset{i}{\sum }\text{P}\left(incidence,Age_{i}\right)}, \end{array}$$

where P(*incidece* | *Age*_*i*_) is the incidence risk of age group *i* and *P*(*Age*_*i*_) is the proportion of age group *i* in the general public. We assumed that the incidence risk for different age groups could be obtained from a logistic function of age, $\text{P}\left(incidence|Age_{i}\right)=1/\left[1+\exp\left(\frac{\mu -i}{r}\right)\right]$. The likelihood of observation for age composition of 5,319 COVID-19 cases can be maximized by searching for optimized μ and γ. Consequently, the incidence risk can be achieved in a maximum likelihood approach where the risk is given by a logistic function of age with estimated parameters μ and γ.

### Assessing Mortality Risk of COVID-19

To assess the mortality risk of COVID-19 in the general public, we used a maximum likelihood approach that is similar to that mentioned above. We obtained the age composition of fatal cases of COVID-19 as

$$\begin{array}{l} \text{P}\left(Age_{i}|Infection,Mortality\right)\\ =\frac{P\left(Mortality|Infection,Age_{i}\right)P\left(Age_{i}|Infection\right)}{\underset{i}{\sum }P\left(Mortality,Age_{i}|Infection\right)}, \end{array}$$

Where *P*(*Mortality* | *Infection, Age*_*i*_) is the risk of mortality condition on an individual's age and infection state and *P*(*Age*_*i*_ | *Infection, Mortality*) is the age composition of fatal cases of COVID-19. In this study, we assumed that infection happens in all age groups for the general public and therefore have *P*(*Age*_*i*_ | *Infection*) = *P*(*Age*_*i*_). We further applied the maximum likelihood approach to obtain the mortality risk of COVID-19 for different age groups in the general public *P*(*Mortality* | *Infection, Age*_*i*_). In the maximum likelihood approach, the mortality risk was given by the aforementioned logistic function of age but with mortality-specific μ and γ. To eliminate the concern that the high mortality risk of older people may inflate the mortality rate of the infected population, we further imputed the mortality rate of the infected people as,

$$\begin{array}{l} P\left(Mortality|Infection\right)=\underset{i}{\sum }P\left(Mortality,Age_{i}|Infection\right) \end{array}$$

## Results

### Characteristics of the COVID-19 Cases and General Public

The public data of a total of 5,319 identified COVID-19 cases were included in our analysis. There were 2,829 (53.2%) males and 2,490 (46.8%) females in the COVID-19 cases; the male to female ratio turned out roughly equal across all age groups. The age of COVID-19 patients ranged from 0.5 to 97 years, with a mean of 45.2 years. The age and gender composition of COVID-19 patients and the public reference are presented in Table 1. Compared to the general public, the COVID-19 cases had a higher average age, and there was a higher proportion of people aged 30–69 years.

**Table 1:** Age and gender composition of the general public and the identified COVID-19 cases.

	**General public**						**COVID-19 cases**					
**Age groups (year)**	**Total**	**Male**	**Female**	**Total (%)**	**Male (%)**	**Female (%)**	**Total**	**Male**	**Female**	**Total (%)**	**Male (%)**	**Female (%)**
0–4	67,393	35,887	31,506	5.89	3.14	2.75	51	24	27	0.96	0.45	0.51
5–9	63,322	34,279	29,043	5.53	2.99	2.54	59	35	24	1.11	0.66	0.45
10–14	62,248	33,775	28,473	5.44	2.95	2 49	55	32	23	1.03	0.60	0.43
15–19	58,258	31,552	26,706	5.09	2.76	2.33	95	55	40	1.79	1.03	0.75
20–24	68,050	36,085	31,965	5.95	3.15	2.79	239	140	99	4.49	2.63	1.86
25–29	92,977	47,710	45,268	8.12	4.17	3.95	356	204	152	6.69	3.84	2.86
30–34	93,201	46,843	46,358	8.14	4.09	4.05	524	291	233	9.85	5.47	4.38
35–39	81,886	41,517	40,370	7.15	3.63	3.53	567	305	262	10.66	5.73	4.93
40–44	83,574	42,557	41,017	7.30	3.72	3.58	579	349	230	10.89	6.56	4.32
45–49	102,384	52,108	50,276	8.94	4.55	4.39	662	354	308	12.45	6.66	5.79
50–54	96,850	48,939	47,911	8.46	4.28	4.19	631	319	312	11.86	6.00	5.87
55–59	69,844	35,208	34,636	6.1	3.08	3.03	494	240	254	9.29	4.51	4.78
60–64	68,014	34,092	33,923	5.94	2.98	2.96	349	157	192	6.56	2.95	3.61
65–69	54,799	26,974	27,825	4.79	2.36	2 43	311	147	164	5.85	2.76	3.08
70–74	34,810	16,905	17,905	3.04	1 48	1.56	153	85	68	2.88	1.60	1.28
75–79	22,799	10,745	12,054	1.99	0.94	1.05	96	46	50	1.80	0.86	0.94
80–84	14,845	6,457	8,389	1.3	0.56	0.73	57	25	32	1.07	0.47	0.60
85–89	6,902	2,870	4,033	0.6	0.25	0.35	28	14	14	0.53	0.26	0.26
90–94	2,031	665	1,365	0.18	0.06	0.12	10	7	3	0.19	0.13	0.06
95+	458	131	327	0.04	0.01	0.03	3	0	3	0.06	0.00	0.06
Total	1,144,648	585,299	559,349	100	51.13	48.87	5,319	2,829	2,490	100	53.19	46.81

**Figure 1:**
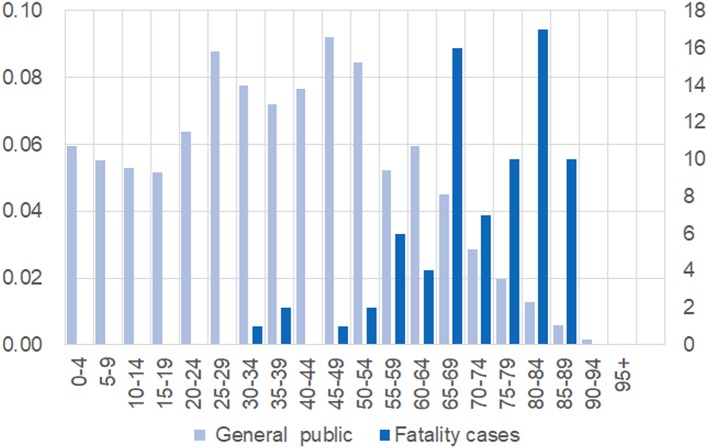
Different age compositions between the general public and 76 fatal cases. Individuals were grouped and presented on the x-axis. For the general public, the proportion of each age group is shown on left-hand side; the number of fatal cases in each group is shown on the right-hand side.

We collected detailed information on 76 fatal cases and plotted the age composition of the cases and the general public in Figure 1. It is evident that death occurs more frequently in older people but is rare for patients under 40 years old. The fatal cases were from 34 to 89 years old, with an average age of 71.47 and a standard deviation of 12.49.

### Incidence Risk of COVID-19 of the General Public

**Figure 2:**
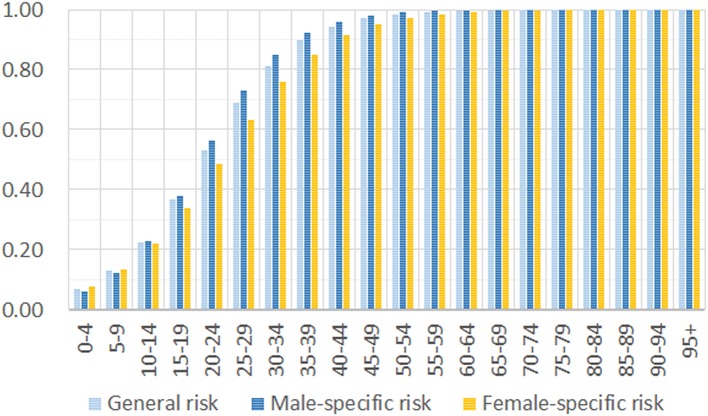
Incidence risk increases in older groups of the general public.

Based on the age composition of 5,319 COVID-19 cases and the 1,144,648 individuals of the general public, we estimated the incidence risk by a maximum likelihood approach. Our results show that the disease can occur in all age groups, and there is no significant difference between males and females (Figure 2). The difference in incidence risk for different genders is observed only for the groups between 15 and 50 years old. After the age of 15 years, males have a slightly higher incidence risk than women, but the increase is negligible for people over 50 years old. Our result does not support a previous report that SARS-CoV-2 generally affects more males than females in the epidemic (8). The incidence risk is low for children and teenagers but rapidly increases for adults. For adults over 40 years of age, the risk is higher than 0.9 when they have full exposure to the virus.

### Mortality Risk of COVID-19 of the General Public

**Figure 3:**
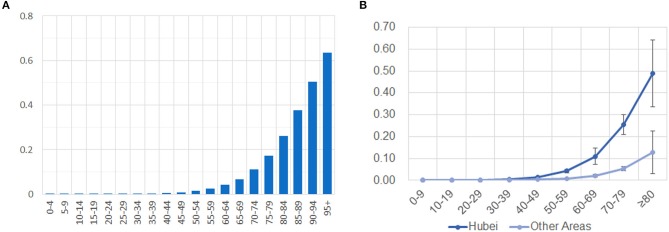
Different mortality risks in different age groups and different parts of China. **(A)** Mortality risks of all fatal cases dramatically increases in older age groups. **(B)** Mortality risks are significantly different in Hubei and other areas of China. The risk is present on the y-axis, while the ages of grouped cases are shown on the x-axis.

In our assessment of mortality risk, there is a significantly higher mortality risk in older adults (Figure 3A). The estimated fatal probability is <0.01 for individuals under 40 years, but it is more than 0.51 for people older than 90 years. The calculated risk is much higher than previous reports stated. Our result is consistent with most of the earlier studies, supporting the hypothesis that older age is associated with an increased risk of mortality in COVID-19 patients. Our analysis of the total of 76 fatal cases suggests a mortality rate of 2.38% for general infection. However, we noticed that the mortality rate of COVID-19 in reports is significantly different between identified cases of Hubei province and that of other parts of China (11).

### Different Mortality Risks in Hubei and Other Provinces

To compare the mortality risk between Hubei and other provinces of China, we divided 76 fatal cases into two subsets: 45 cases from Hubei province and 31 cases from other parts of China. The aforementioned statistical analysis for mortality risk was applied to the two subsets, with nine different age groups in each. To account for variability, we further obtained a standard deviation of estimates by applying the same method to 1,000 simulated data sets that were generated from the initial estimation. Our results show that mortality risk is no more than 0.13 ± 0.10 for people over 80 years outside Hubei province, but the risk is as high as 0.60 ± 0.15 for the corresponding age group in Hubei province (Figure 3B). Mortality risk falls under 0.05 for people younger than 70 years in other parts of China, while only people under 50 years have a risk under 0.05 in Hubei province. We also calculated the expectation of mortality rates for a general infection inside and outside Hubei province as 4.78 and 0.95%, respectively.

## Discussion

Our results suggest that there is a significantly higher mortality risk for COVID-19 in seniors than that given in the previous report (11). This is probably due to the published report not accounting for the increasing death rate of the identified COVID-19 cases. In the previous study, the crude mortality rate was only 2.3%, but the rate was 3.7% on March 4, 2020 (3015 deaths among 80,566 identified COVID-19 cases). As of March 4, 2020, there were still 5,952 COVID-19 patients in critical condition in China. It has been reported that the survival probability of critically ill patients continuously decreased with the increase of time since admission to the intensive care unit (12). Our analysis was based on the composition of the age of the different populations, and therefore it is less affected by the disease progression of patients, especially the increasing death rate of critically ill patients. Age has been reported as the independent predictor of an adverse outcome in SARS and MERS. Comorbidities and low immune function in older people might be the major cause of a higher mortality of coronaviruses (3, 4, 11). Prompt administration of antibiotics to prevent infection and the strengthening of immune support treatment might reduce the mortality of seniors (8).

Our data showed that the mortality rate of COVID-19 is five times higher in Hubei province than that in other parts of China. This result is supported by an early publication that stated the estimated case fatality rate of mainland China excluding Hubei was 0.15%, far less than that of the Hubei province excluding the city of Wuhan (1.41%) and Wuhan city (5.25%) (13). On the one hand, this difference may be partially explained by insufficient medical resources due to such a large number of patients in Hubei Province during the outbreak. According to the 2018 annual brief report of the health service development in Wuhan city, there were 8.6 hospital beds per 1,000 people. However, hospital bed utilization ratio of 2017 and 2018 reached 92.34 and 94.22%, respectively (14). It was shown that, even under normal circumstances, there were few spare beds. On the other hand, detailed information on the majority of fatal cases (40 of 45 in total) from Hubei province was published before January 25, 2019. The mortality rate of early reported cases may be overstated, because case detection is highly biased toward the more severe cases. However, we strongly suggest that international authorities try their best to immediately prevent COVID-19 patients from overloading their health care system. Our hypothesis that a smooth-running health care system can effectively reduce the mortality rate of COVID-19 is strongly supported by the low mortality rate in other parts of China.

In conclusion, we investigated the incidence and mortality risks of the infection by analyzing the age composition of COVID-19 patients and the general public in China. Our data show a relatively low incidence risk for young people but a very high mortality risk for older adults. Therefore, it is prudent to strengthen the tertiary preventive and clinical care of old-aged patients to reduce mortality. Furthermore, our results also support the conclusion that a good medical service can effectively reduce the mortality rate of the viral infection to 1% or less. Our study could be of value to medical authorities to implement effective medical service. The lack of complete data for all COVID-19 cases potentially increases the occurrence of selection and measurement biases in this study. Therefore, further large-scale epidemiological studies are necessary to elucidate the risk factors of COVID-19 for the general public.

## Data Availability Statement

All datasets generated for this study are available upon request.

## Ethics Statement

Ethical review and approval was not required for the study on human participants in accordance with the local legislation and institutional requirements. Written informed consent from the participants' legal guardian/next of kin was not required to participate in this study in accordance with the national legislation and the institutional requirements.

## Author Contributions

YH, HW, and LL contributed to the conception and design of the study. HL, SW, WB, and YL collected and analyzed the data. YH, HL, and FZ wrote sections of the manuscript. All authors contributed to manuscript revision and have read and approved the submitted version.

## Conflict of Interest

The authors declare that the research was conducted in the absence of any commercial or financial relationships that could be construed as a potential conflict of interest.
